# Effects of Formulation and Processing Variables on the Rheology of Chitosan–Vanillin-Stabilized Olive Oil–Water Emulsions for Oleogel Applications

**DOI:** 10.3390/foods15071233

**Published:** 2026-04-04

**Authors:** Leticia Montes, David Rey, Ramón Moreira, Daniel Franco

**Affiliations:** Department of Chemical Engineering, Universidade de Santiago de Compostela, Rúa Lope Gómez de Marzoa, s/n, 15782 Santiago de Compostela, Spain; leticia.montes.martinez@usc.es (L.M.); david.rey.nogueira@rai.usc.es (D.R.); ramon.moreira@usc.es (R.M.)

**Keywords:** chitosan–vanillin oleogels, interfacial structuring, Herschel–Bulkley rheology, Schiff base crosslinking, yield stress, thermal stability

## Abstract

The rheological behavior of chitosan–vanillin crosslinked olive oil-in-water emulsions (Φ = 0.52) was investigated to identify formulation and processing conditions suitable for designing oleogel precursors. The effects of homogenization conditions, reaction temperature, chitosan concentration, vanillin-to-chitosan molar ratio, and non-ionic surfactants were systematically evaluated. Surfactant-free emulsions exhibited a structured, gel-like response and non-thixotropic shear-thinning flow, which was well described by the Herschel–Bulkley model within the investigated shear-rate range. Optimal homogenization (4 min, ≥9500 rpm) refined the microstructure without compromising stability. Increasing the reaction temperature to 55 °C, the chitosan concentration to ~0.9% (*w*/*w*), and the vanillin-to-chitosan molar ratio to 0.7 maximized yield stress, consistency, and thermal robustness, consistent with enhanced network formation. In contrast, Tween^®^ surfactants produced divergent responses, increasing small-amplitude oscillatory stiffness while markedly reducing resistance under steady shear, likely due to surfactant-driven interfacial displacement. Among the tested surfactants, Tween^®^ 20 provided the highest thermal stability. Overall, these results define processing and formulation windows to obtain surfactant-free, structured emulsions with improved structuring performance, supporting their use as effective templates for olive oil oleogel development.

## 1. Introduction

The replacement of saturated (SFA) and trans fats (TFA) with healthier alternatives has become a critical priority in food innovation due to the well-established relationship between these types of fats and the increased risk of cardiovascular and metabolic diseases. SFA, mostly found in animal products, and TFA, often present in processed foods, are linked to elevated cholesterol levels and systemic inflammation that contribute to chronic health conditions [1]. Vegetable oils, which are predominantly liquid at room temperature, usually offer beneficial fatty acid compositions, especially rich in mono- and polyunsaturated fatty acids with positive effects on human metabolism [2]. However, their liquid state limits the direct use as replacements for solid animal fats in products that require specific textures and stability. Consequently, the structuring of liquid vegetable oils into semi-solid networks resembling animal fats has attracted great scientific and industrial interest, boosting the development of structuring strategies to convert liquid oils into semi-solid systems, such as oleogels, that resemble animal fats in technological performance. Oleogels are emerging semi-solid systems in which liquid vegetable oils are immobilized within a three-dimensional network formed by structuring agents such as polymers or low-molecular-weight compounds [3]. These networks impart mechanical stability while mimicking the texture and mouthfeel of conventional solid fats. Among the different structuring strategies, the emulsion-template approach has gained considerable attention due to its versatility in structuring oils using hydrophilic biopolymers, including polysaccharides and proteins [4]. In this method, an oil-in-water emulsion is first prepared with the structuring agent dissolved in the aqueous phase; subsequent removal of water by drying yields a continuous solid matrix that entraps the oil droplets within the polymeric network [3,5]. Consequently, the microstructural features of the initial emulsion—such as droplet size, spatial organization, and stability—play a decisive role in determining the properties of the resulting oleogel. The formation of stable emulsion templates, however, remains technically challenging, as destabilization phenomena including coalescence, creaming, or phase separation must be carefully controlled to preserve structural integrity [6]. Emulsion structure is governed by a complex interplay of processing parameters (e.g., homogenization intensity and duration, thermal history) and formulation variables (e.g., gelator concentration, oil volume fraction, component ratios, and the presence of surfactants or emulsifiers). Because the final oleogel largely inherits the droplet packing and continuous-phase connectivity established in the parent emulsion, a rigorous evaluation of how these variables affect emulsion structure is essential. In this context, rheology provides a particularly powerful and sensitive tool, as it quantitatively reflects microstructural organization and has been proposed as a predictive descriptor for the manufacture and performance of highly structured emulsions [7]. Nevertheless, despite the growing interest in emulsion-templated oleogels, systematic studies linking processing and formulation variables to emulsion rheology, and subsequently to oleogel properties after dehydration, remain scarce. Instead, many studies rely on processing conditions adopted from previous work, often without rheological justification for their suitability in specific biopolymer–surfactant systems, thereby limiting reproducibility and rational scale-up. Addressing this gap through controlled, multivariable rheological investigations is therefore crucial for the rational design of emulsion templates capable of yielding robust and reproducible oleogels.

Chitosan, a bio-based polysaccharide derived from chitin found abundantly in crustacean shells, offers an attractive option as a structuring agent due to its biodegradability and biocompatibility [8]. Vanillin, widely used industrially as a flavoring agent, possesses functional groups capable of forming covalent and hydrogen bonds with chitosan, thus reinforcing the polymeric network [9]. Specifically, recent literature on chitosan-based oleogels reveals substantial variability in the conditions used to prepare the initial emulsions. Most studies employ high-shear mechanical homogenization—typically using Ultra-Turrax or equivalent devices—to generate oil-in-water (O/W) emulsions, with reported homogenization times ranging from 2 to 4 min and rotational speeds between 9000 and 15,000 rpm [10,11,12,13,14,15] or even milder conditions such as 30 min and 600 rpm [16]. Such differences might have important implications for reproducibility and represent a major limitation for translating these systems into larger-scale processes. Emulsification is generally performed under mild, non-thermal conditions; however, temperature is a critical factor in the Schiff base reaction, and its effects on emulsion stability remain unexplored. Moreover, nearly all reported studies use freeze-drying for the dehydration step (typically at −40 to −60 °C), the only exception being Lin et al. [10]. Regarding compositional variables, chitosan concentration is the most consistently reported parameter, as it is widely recognized as a key structuring factor. Nonetheless, chitosan-based formulations are typically restricted to a narrow concentration window (~0.7–3% *w*/*v*), and different studies use non-standardized units, making direct comparisons difficult without additional assumptions or recalculations. Vanillin is universally described as a Schiff base crosslinker because crosslinking is central to oleogel formation and stability; yet none of the available studies explicitly reports the vanillin to chitosan molar ratio. Notably, at least two studies [10,13] did not rely on the Schiff base reaction, but instead achieved structuring using glycyrrhizic acid [10] or whey protein isolate [13]. Although the Schiff base reaction between the primary amines of chitosan and the aldehyde group of vanillin yields a crosslinked structure that enhances the mechanical and functional properties of the system [12], such methodological variability in the literature precludes a mechanistic comparison of crosslinking density, thereby constituting a significant knowledge gap, particularly for establishing structure–property relationships. Finally, some of the aforementioned studies rely on biopolymer-based interfacial stabilization, while others selectively incorporate Tween^®^ 60 to enhance emulsification efficiency.

Overall, emulsification is treated as a preparative step rather than a controlled process variable. Consequently, key operation variables—such as homogenization time, shear rate, temperature, and molar ratios—are systematically underreported, despite their known influence on droplet size, interfacial organization, and the final structure of the oleogel. Therefore, this study investigates olive oil–water emulsions stabilized by chitosan and vanillin as an intermediate material in oleogel production, to systematically evaluate the influence of critical operational variables on the emulsion rheological and mechanical properties. Based on this analysis, an optimized protocol is proposed to obtain reproducible emulsions with strong potential for application as healthier, functional fat replacers in food formulations.

## 2. Materials and Methods

### 2.1. Materials

Medium-molecular-weight chitosan was purchased from Sigma-Aldrich (St. Louis, MO, USA). The degree of deacetylation was 75–85%, and the viscosimetric molecular weight was 300 ± 18 Kg/mol. Food-grade olive oil was supplied by Aceites Abril SL (Ourense, Spain). Vanillin (152.15 g/mol), Tween^®^ 20 and Tween^®^ 60 were purchased from Sigma-Aldrich and used as received.

### 2.2. Preparation of Chitosan and Vanillin Solutions

Chitosan was dissolved in a 1% (*v*/*v*) acetic acid aqueous solution and stirred at 400 rpm at 50 °C for 2 h. pH was adjusted to 4.5 with sodium acetate buffer. Solutions were prepared at different concentrations (0.6% to 1.0% *w*/*v*). These chitosan concentrations were selected based on the desired final chitosan concentration within the emulsion, namely 0.6, 0.7, 0.8, 0.9, and 1.0% (*w*/*w*), corresponding to initial stock solution concentrations of 1.33, 1.55, 1.78, 2.00, and 2.22% (*w*/*w*), respectively. After 2 h, the chitosan solutions were vacuum-filtered using a Kitasato flask and filter paper to remove insoluble solids that had not completely dissolved. Ethanol was used to dissolve vanillin due to its low solubility in water. The preparation procedure consisted of weighing the desired amount of vanillin using an analytical balance, followed by the addition of 91% (*w*/*w*) ethanol. The ethanol concentration was determined using a refractometer (ATAGO, ABBE DR-A1, Tokyo, Japan). The solutions were stirred under magnetic agitation for 30 min using a magnetic stirrer. Once both solutions were prepared, the emulsion was subsequently formed. Emulsions were prepared with a 50:50 (*w*/*w*) oil-to-aqueous phase composition. For each formulation, 50 g of emulsion was produced. Specifically, 2.5 g of vanillin solution, 22.5 g of chitosan solution, and 25 g of oil were weighed using a precision balance (Germini BV, Mettler PJ3000, The Netherlands).

### 2.3. Emulsion Preparation

Olive oil was added dropwise via burette directly through the Ultra-Turrax probe (IKA T25 basic, Staufen im Breisgau, Germany) at the specified homogenization speeds (6500, 9500, 13,500, and 17,500 rpm) to the chitosan solution, previously prepared at 0.6–0.9% *w*/*v*, (Section 2.2) placed on an orbital shaker (P-Selecta Rotaterm, Barcelona, Spain) at 150 rpm to promote homogeneous mixing of the emulsion components from the beginning and to minimize the formation of dead zones. Addition occurred over 3 min, followed by a 30 s pause. Vanillin was added over the next 30 s. Secondary homogenization continued for specified times (0.5, 2, 4, or 10 min) at constant speed, with the beaker immersed in a cold-water bath for temperature control for viscous samples or extended processing. Post-homogenization, the emulsion was rapidly transferred to a closed vessel and subjected to magnetic stirring (400 rpm) for 2 h at different reaction temperatures (25, 40, or 55 °C) to promote chitosan–vanillin cross-linking. After this reaction period, the emulsion was allowed to mature at room temperature for an additional 22 h prior to analysis, ensuring completion of the cross-linking process.

Oil-to-aqueous addition order proved critical, as reverse addition yielded phase-separated systems; the adopted oil-into-chitosan protocol followed Brito et al. [12]. Tween^®^ 20/60 variants incorporated 0.4% *w*/*w* surfactant directly into the chitosan solution prior to identical emulsification. All formulations maintained constant oil phase volume (ϕ ≈ 0.52) across experimental variables. Oil volume fraction (ϕ) is calculated as the ratio between the volume of the oil phase (*V*_oil_) and the total volume (*V*_total_) of the emulsion:

(1) $$\phi =\frac{V_{\mathrm{oil}}}{V_{\mathrm{total}}}\;\mathrm{with}V_{\mathrm{total}}=V_{\mathrm{oil}}+V_{\mathrm{aqueous}}$$

As the formulation is prepared gravimetrically, volumes are obtained from masses (*m*_i_) using the corresponding densities (*ρ*_i_):

(2) $$V_{i}=\frac{m_{i}}{\rho_{i}}\Rightarrow \phi =\frac{m_{\mathrm{oil}}/\rho_{\mathrm{oil}}}{\left(m_{\mathrm{oil}}/\rho_{\mathrm{oil}})+(m_{\mathrm{aqueous}}/\rho_{\mathrm{aqueous}}\right)}$$

### 2.4. Experimental Design

The effect of processing and compositional variables on emulsion rheology and stability was investigated using a systematic experimental design. The factors considered were homogenization speed and time, emulsification temperature, chitosan concentration, vanillin-to-chitosan molar ratio and emulsifier type/level (Tween^®^ 20 or Tween^®^ 60). The specific levels tested for each factor are summarized in Table 1.

The study was conducted as a one-factor-at-a-time (OFAT) screening rather than a factorial design. As summarized in Table 1, homogenization time (0.5–10 min), homogenization speed (6500–17,500 rpm), reaction temperature (25–55 °C), chitosan concentration (0.6–1.0% *w*/*v*), vanillin-to-chitosan molar ratio (0.0–1.3), and emulsifier type (Tween^®^ 20 vs. Tween^®^ 60) were varied independently while the remaining variables were kept constant at the reference conditions. Therefore, factor–factor interactions were not systematically evaluated, and no full factorial statistical analysis was performed.

### 2.5. Rheological Characterization and Emulsion Stability

Rheological properties were determined using a controlled-stress rheometer (Anton Paar Physica MCR 301, Anton Paar, Austria) with 50 mm parallel-plate geometry and 1.5 mm fixed gap. Samples were loaded carefully, edges sealed with paraffin to prevent evaporation, and measurements conducted at 25 °C. All measurements were controlled using RheoPlus software (v3.21, Anton Paar) for rheometer operation as well as real-time data acquisition and visualization. Prior to evaluation (24 h after preparation), the emulsions were homogenized for several minutes using a magnetic stirrer at room temperature and 400 rpm. A volume of emulsion was carefully transferred using a pipette and placed at the center of the lower plate of the rheometer. The upper plate was lowered and adjusted to a fixed gap of 1.5 mm, which was maintained constantly. Excess sample extruded at the edges was carefully removed with tissue paper, and the rim was sealed with paraffin to prevent solvent evaporation (water or ethanol), which could otherwise affect the results. Additionally, a plastic solvent trap was placed over the measuring geometry to further isolate the testing area.

After loading, the sample was allowed to rest for 15 min prior to testing to enable structural relaxation and to avoid measuring recovery stresses generated during plate positioning. The test sequence consisted of time sweep, determination of the linear viscoelastic region (strain sweep), mechanical spectrum (frequency sweep), flow curve, temperature ramps, and subsequent determination of the linear viscoelastic region and mechanical spectrum after the temperature ramps. All measurements were conducted at 25 °C, except for the temperature ramp tests. Temperature was controlled using a Peltier system with a precision of ±0.01 °C.

#### 2.5.1. Time Sweeps

Time sweeps were performed at constant shear rate of 10 s^−1^ for 8 min to assess time-dependent behavior (thixotropy/rheopexy), confirm system stability, and verify completion of chitosan–vanillin reaction prior to subsequent tests.

#### 2.5.2. Strain Sweeps

Strain sweeps were conducted at 1 Hz over 0.01–1000% strain to determine the linear viscoelastic region (LVR). Storage (G′) and loss (G″) moduli remained constant below 1% strain, which was selected for all subsequent oscillatory tests.

#### 2.5.3. Frequency Sweeps

Frequency sweeps were performed at 1% strain over 0.01–100 Hz at 25 °C to characterize viscoelastic properties and microstructural features under different processing conditions.

#### 2.5.4. Flow Curves

Steady shear measurements were determined at different shear rates from 0.01–100 s^−1^. Apparent viscosity data were fitted to the Herschel–Bulkley model to evaluate effects of formulation and processing variables on flow behavior. Data were fitted to the Herschel–Bulkley model, shown in Equation (3):

(3) $$\tau \;=\;\tau_{0}+K{\dot{\gamma }}^{n}$$

where *τ*_0_ is the yield stress (Pa), *K* is the consistency index (Pa·s^n^), and *n* is the flow behavior index (dimensionless).

#### 2.5.5. Temperature Ramps

Temperature ramps (1 Hz, 1% strain) were used to assess thermal stability in selected formulations, monitoring the evolution of complex viscosity (η*) throughout the thermal cycle. Complex viscosity was calculated from the complex shear modulus as η* = G*/ω, where G* is the complex modulus obtained from oscillatory measurements and ω is the angular frequency (rad·s^−1^).

The following temperature cycles were used: 10 min at 25 °C, heating 25–50 °C (3 °C min^−1^), 10 min at 50 °C, cooling 50–25 °C (3 °C min^−1^) and 10 min at 25 °C.

### 2.6. Statistical Analysis

All experiments were conducted in duplicate, and results are reported as mean ± standard deviation. One-way analysis of variance (ANOVA) was performed using SPSS Statistics 29 (IBM Corp., Chicago, IL, USA) to assess the effects of independent variables on dependent responses, followed by Duncan’s post-hoc test for mean separation at a significance level of *p* < 0.05. Because the experimental plan (Table 1) followed an OFAT screening approach rather than a full factorial design, factor–factor interactions were not assessed, and thus potential interaction effects cannot be ruled out. Model goodness-of-fit was evaluated using the coefficient of determination (R^2^) and root mean square error (*RMSE*), calculated as, Equation (4):

(4) $$RMSE=\sqrt{\frac{\sum {\left(X_{exp}-X_{calc}\right)}^{2}}{N-P}}$$

where *X_exp_* and *X_calc_* represent experimental and calculated values, respectively, *N* is the number of data points, and *P* is the number of model parameters.

## 3. Results and Discussion

### 3.1. Time Sweep

Time sweep tests were performed at a constant shear rate of 10 s^−1^ to evaluate the rheological stability of the emulsions under continuous deformation, to detect possible time-dependent effects (thixotropy or rheopexy), and to verify that no significant structural rearrangements occurred during the measurement time. In addition, these tests served to confirm that the chitosan–vanillin reaction had effectively reached completion prior to subsequent rheological characterization.

Across all formulations and processing conditions studied—including variations in homogenization time and speed, reaction temperature, chitosan concentration, vanillin-to-chitosan molar ratio, and the presence of non-ionic surfactants—all emulsions exhibited stable viscosity profiles throughout the duration of the test (8 min). No significant changes in apparent viscosity were observed with time, indicating the absence of thixotropic or rheopectic behavior within the applied shear-rate range. This confirms that the emulsions behaved as time-independent, non-thixotropic systems under steady shear, and that the rheological responses measured in subsequent tests were not influenced by ongoing structural evolution during the experiments.

Although apparent viscosity remained constant over time for all systems, its absolute magnitude depended strongly on formulation and processing variables. Increasing homogenization time (from 0.5 to 10 min) and speed (from 6500 up to 17,500 rpm) increased apparent viscosity from 2.6 to 3.3 Pa s and from 2.6 to 4.2 Pa s, respectively. which might be attributed to droplet size reduction and enhanced hydrodynamic interactions within the emulsion, as previously described for mechanically homogenized biopolymer-stabilized emulsions [17]. These observations are consistent with the well-established relationship between shear intensity, droplet breakup, and flow resistance in concentrated dispersions [18,19].

Reaction temperature exerted a significant influence on the apparent viscosity of the emulsions. Emulsions reacted at higher temperatures are expected to exhibit systematically higher apparent viscosity values due to accelerated chitosan–vanillin Schiff base formation and the consequent development of a more interconnected droplet network. Reaction temperature also exerted a clear influence on apparent viscosity. Emulsions reacted at higher temperatures exhibited systematically higher apparent viscosity values (from 2.9 Pa·s at 25 °C up to 4.2 Pa·s at 55 °C), reflecting accelerated chitosan–vanillin Schiff base formation and the development of a more interconnected droplet network. Similar temperature-driven enhancements in emulsion structuring have been reported for polysaccharide-based systems undergoing thermally activated interactions [20].

The reaction between vanillin and chitosan has been extensively studied, and its mechanism is well established. In this regard, Brito et al. [12] demonstrated the occurrence of Schiff-base (C=N) crosslinking between both compounds through converging evidence obtained from FTIR spectroscopy, together with structural and microstructural analyses. This comprehensive approach confirmed the formation of a chitosan–vanillin network rather than relying on a single analytical observation. Moreover, the development of a more compact network cannot be attributed solely to covalent Schiff-base formation. It is also likely reinforced by secondary interactions, particularly hydrogen bonding involving residual free functional groups. After reacting with chitosan via the primary Schiff-base mechanism, vanillin retains hydroxyl and methoxy groups capable of forming hydrogen bonds either with other vanillin molecules or with remaining amino groups on chitosan, thereby enhancing network cohesion and compactness [21].

Regarding the effect of temperature, Marin et al. [22] reported that, under anhydrous conditions, Schiff-base reactions reach maximum conversion after approximately two hours, with reaction progress being further promoted at elevated temperatures. Collectively, these findings support the idea that increasing temperature can accelerate the kinetics of chitosan–vanillin crosslinking while also favoring non-covalent interactions, ultimately contributing to the formation of a more organized and mechanically robust network.

Chitosan concentration had the most pronounced effect on apparent viscosity magnitude (from 2.5 Pa·s at 0.6% up to 7.4 Pa·s at 0.9%). A clear separation was observed between low-concentration systems (≤0.7% *w*/*w*) and high-concentration systems (≥0.8% *w*/*w*), with the latter exhibiting substantially higher viscosity. This trend may be related to the formation of a denser continuous-phase network and enhanced polymer-mediated droplet interactions at higher biopolymer loadings, as previously reported for chitosan- and gum-based emulsions [20,23,24,25].

The vanillin-to-chitosan molar ratio also influenced emulsion viscosity, with intermediate ratios leading to higher apparent viscosity than formulations prepared either in the absence of vanillin or with excess vanillin. In particular, the system at a molar ratio of 0.7 exhibited the highest apparent viscosity (~3.3 Pa·s), whereas lower ratios (0.3 or 0.0) and excess vanillin (1.3) resulted in comparatively lower values.

This trend suggests that an optimal stoichiometric balance between vanillin aldehyde groups and chitosan amino groups promotes the formation of a more effective cross-linked network through Schiff base reactions [12]. When this balance is not achieved, crosslinking efficiency is likely reduced, either because of an insufficient number of reactive groups at low vanillin contents or because excess, unreacted vanillin may interfere with polymer–polymer interactions at the interface. Overall, these findings indicate that the increase in viscosity depends not only on the presence of vanillin, but also on the stoichiometric balance established during network formation. Similar stoichiometry-dependent structuring effects have been reported in other aldehyde-crosslinked polysaccharide systems [21].

In contrast, the incorporation of non-ionic surfactants (Tween^®^ 20 and Tween^®^ 60) led to a marked reduction in apparent viscosity compared to surfactant-free emulsions (from 2.9 Pa·s without surfactant up to 2.3 Pa·s with both surfactants), despite their known ability to reduce droplet size and enhance emulsification efficiency. This effect might be attributed to the competitive adsorption of surfactant molecules at the oil–water interface, which partially displaces chitosan–vanillin interfacial networks and weakens the load-bearing structure under steady shear, as described in previous studies on mixed biopolymer–surfactant systems [19,26].

Overall, time sweep results demonstrate that all emulsions were kinetically stable during rheological testing and that the differences observed in subsequent oscillatory and steady shear measurements arise from intrinsic microstructural variations induced by formulation and processing variables, rather than from time-dependent effects or incomplete network formation. A long-term emulsion stability is not strictly required in this case, because the system will be dried shortly after preparation (within ~24 h). During this short interval and especially throughout the drying step, the Schiff-base reaction is expected to continue and even be promoted, as previously reported [22]. In fact, Karaer and Kaya [27] used scanning electron microscopy to show that products formed via Schiff-base reactions can display a porous, layered architecture with a relatively smooth surface.

### 3.2. Strain Sweep and Determination of the Linear Viscoelastic Region

Strain sweep tests were conducted at a constant frequency of 1 Hz to determine the limits of the linear viscoelastic region (LVR) and to establish an appropriate strain amplitude for subsequent oscillatory measurements. Identifying the LVR is essential to ensure that the viscoelastic moduli reflect the undisturbed microstructure of the emulsions, without inducing irreversible structural breakdown [28].

For all emulsions investigated, irrespective of processing conditions or formulation variables, both the storage modulus (G′) and the loss modulus (G″) remained constant over a wide range of strain amplitudes. Within this linear region, G″ values were consistently higher than G′ for all formulations, indicating predominantly viscous behavior. No significant deviations from linear viscoelastic behavior were observed up to strains of approximately 1%, indicating that the emulsions possessed a sufficiently robust structure to withstand small deformations without network disruption. Beyond this limit, a progressive decrease in G′ was observed, suggesting the onset of structural breakdown and rearrangement of the droplet–polymer network. Based on these results, a strain amplitude of 1% was selected for all subsequent frequency sweep and temperature ramp measurements.

Although the extent of the LVR was comparable across all systems, the absolute values of G′ and G″ varied markedly depending on formulation and processing parameters, with G′ typically ranging from approximately 0.5 to 30 Pa and G″ from about 2 to 100 Pa within the linear viscoelastic region. In general, increases in homogenization time and speed led to higher moduli, suggesting the formation of finer droplet dispersions and enhanced inter-droplet interactions, as previously described for mechanically homogenized emulsions [17,18,19].

Reaction temperature had a more pronounced effect on emulsion viscoelasticity, as samples processed at elevated temperatures exhibited significantly higher moduli. Within the linear viscoelastic region, G′ increased from approximately 8–11 Pa at 25 °C to about 13–15 Pa at 55 °C, while G″ varied more moderately, remaining in the range of 23–28 Pa. These results are consistent with enhanced chitosan–vanillin crosslinking at higher reaction temperatures, likely leading to progressive densification of the interfacial network. The more marked increase in G′ relative to G″ indicates that increasing temperature primarily reinforces the elastic character of the system, consistent with the development of a stronger and more interconnected structure [20].

Chitosan concentration had the most pronounced effect on the viscoelastic response. A clear transition was observed between low-concentration systems (≤0.7% *w*/*w*) and high-concentration systems (≥0.8% *w*/*w*), with the latter showing substantially higher G′ and G″ values. Within the linear viscoelastic region, G′ increased from approximately 8–11 Pa for the lowest concentrations to about 19–22 Pa at higher chitosan contents, while G″ increased from roughly 23–34 Pa to about 47–52 Pa. These results indicate the development of a more interconnected and load-bearing network at higher biopolymer concentrations, reinforcing the gel-like character of the emulsions even within the linear regime. This behavior suggests the onset of a percolated droplet–polymer network at higher biopolymer loadings, resulting in increased resistance to deformation even within the linear regime, as reported for chitosan- and gum-stabilized emulsions [20,23,24,25].

In contrast, changes in the vanillin-to-chitosan molar ratio had a comparatively weaker effect on the viscoelastic moduli. Within the linear viscoelastic region, G′ values for the crosslinked systems ranged from approximately 5 to 11 Pa, whereas G″ remained between about 20 and 30 Pa. Interestingly, the formulation prepared without vanillin displayed markedly higher moduli (G′ ≈ 50–60 Pa; G″ ≈ 110–115 Pa), indicating that vanillin incorporation did not necessarily translate into a stronger viscoelastic response under the conditions evaluated. This behavior may be explained by the greater mobility of non-crosslinked chitosan chains, which could favor intermolecular associations such as hydrogen bonding, chain entanglements, and droplet bridging. By contrast, crosslinking through Schiff base formation may have produced a more compact interfacial layer while simultaneously limiting chain flexibility and reducing the extent of polymer–polymer interactions, resulting in lower viscoelastic moduli. Nevertheless, among the crosslinked systems, formulations prepared near the stoichiometric ratio exhibited slightly higher G′ and G″ values than those prepared either with excess vanillin or with lower aldehyde content. This trend suggests that a balanced availability of aldehyde and amine groups may favor more efficient crosslinking within the polymer network. Comparable stoichiometry-dependent effects have been described in other chitosan–aldehyde crosslinked systems [12].

The addition of non-ionic surfactants (Tween^®^ 20 and Tween^®^ 60) led to an increase in both G′ and G″ compared to surfactant-free emulsions, particularly at low strain amplitudes. Within the linear viscoelastic region, surfactant-free emulsions exhibited G′ values of approximately 8–11 Pa and G″ values of about 23–26 Pa, whereas systems containing Tween^®^ 20 showed G′ ≈ 16–20 Pa and G″ ≈ 28–31 Pa. The highest moduli were observed for Tween^®^ 60, with G′ around 22–27 Pa and G″ about 30–33 Pa. This indicates enhanced elastic contributions under small deformations, likely arising from improved interfacial coverage and reduced droplet size [19,26]. However, as discussed in subsequent sections, this apparent stiffening under oscillatory conditions does not directly translate into improved resistance under steady shear.

Overall, strain sweep measurements confirmed that all emulsions exhibited a well-defined linear viscoelastic region and that the selected strain amplitude was appropriate for comparative analysis. Differences in viscoelastic moduli within the LVR reflect intrinsic microstructural variations induced by formulation and processing variables, providing a reliable basis for interpreting frequency-dependent and steady shear rheological behavior.

### 3.3. Frequency Sweep

Frequency sweep tests were carried out at a constant strain of 1%, within the previously determined linear viscoelastic region, to investigate the frequency-dependent viscoelastic behavior of the emulsions and to gain insight into their microstructural organization and relaxation dynamics. These measurements provide information on the balance between elastic and viscous contributions, as well as on the stability of the droplet network over different deformation time scales [28].

**Figure 1:**
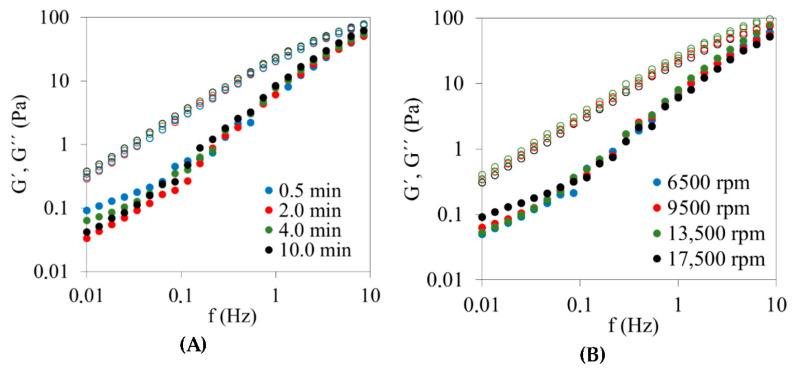

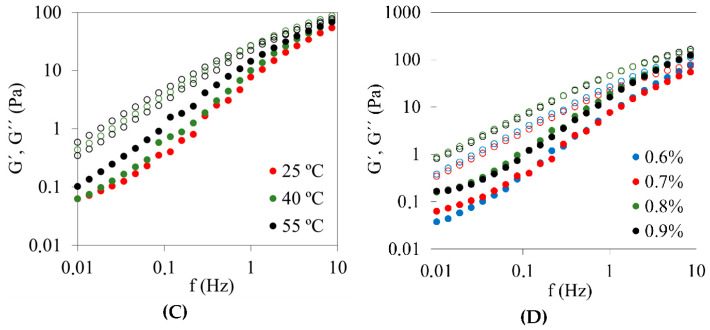

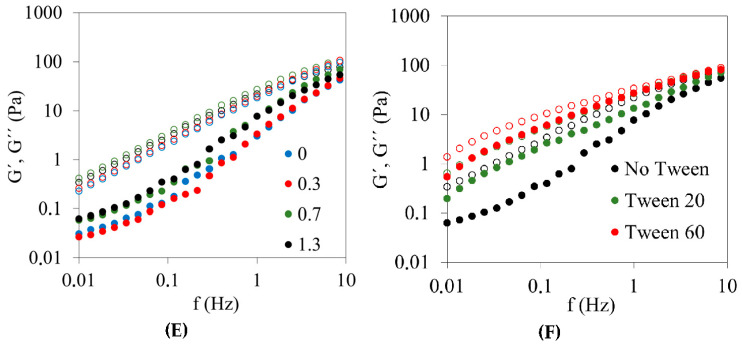
Effect of processing and formulation variables on the frequency-dependent viscoelastic behavior of olive oil–water emulsions. Frequency sweep tests were performed at 1% strain (within the linear viscoelastic region). Storage modulus (G′, closed symbols) and loss modulus (G″, open symbols) are shown. Unless otherwise specified, emulsions were prepared at 13,500 rpm, 25 °C reaction temperature, 0.8% chitosan, and vanillin-to-chitosan molar ratio 0.7. (**A**) Homogenization time (0.5, 2.0, 4.0, and 10.0 min); (**B**) homogenization speed (6500, 9500, 13,500, and 17,500 rpm); (**C**) reaction temperature (25, 40, and 55 °C); (**D**) chitosan concentration (0.6, 0.7, 0.8, and 0.9%); (**E**) vanillin-to-chitosan molar ratio (0, 0.3, 0.7, and 1.3); and (**F**) emulsifier type (no Tween, Tween^®^ 20, and Tween^®^ 60). Storage modulus (G′, closed symbols) and loss modulus (G″, open symbols) are shown.

As shown in Figure 1, in all emulsions studied, the loss modulus (G″) exceeded the storage modulus (G′) over the entire frequency range investigated, indicating a predominantly viscous, liquid-like behavior. This response is characteristic of concentrated oil-in-water emulsions with dispersed phase volume fractions below the jamming threshold [7,28], and is consistent with the fixed oil volume fraction (Φ ≈ 0.52) employed throughout this study. Despite this viscous dominance, all systems exhibited measurable elastic contributions, which might indicate the presence of structured droplet networks rather than simple Newtonian fluids [20,23,24].

A common feature observed in surfactant-free emulsions was a distinct change in the slope of G′ at low frequencies, typically around 0.1 Hz. Below this frequency, G′ increased weakly with frequency, while above it a steeper dependence was observed, leading to a gradual convergence of G′ and G″ at higher frequencies. This behavior suggests the presence of transient droplet associations and relaxation processes associated with inter-droplet interactions mediated by the chitosan–vanillin network [23,29]. The frequency range investigated thus corresponds to a transition regime between viscous-dominated flow at long time scales and increasingly elastic response at shorter time scales [28]. These mechanical spectra indicate that long-term stability is restricted in these emulsions, but this fact is not particularly relevant considering that these emulsions are intermediate materials usually prepared shortly before the production of oleogels.

The effect of homogenization time and speed on the frequency spectra is presented in Figure 1A and Figure 1B, respectively. Increasing homogenization time (0.5–10 min) led to a progressive increase in both G′ and G″ across the frequency range, while preserving the overall spectral shape (Figure 1A). Similarly, higher homogenization speeds (6500–17,500 rpm) resulted in higher absolute moduli without altering the qualitative frequency dependence (Figure 1B).

Overall, processing variables related to homogenization exerted only a minor influence on the frequency-dependent viscoelastic response in terms of spectral shape. While increased shear intensity during emulsification resulted in higher absolute moduli due to droplet size refinement, the overall form of the frequency spectra remained largely unchanged. This indicates that homogenization primarily affects microstructural length scales rather than the fundamental nature of the viscoelastic network once formed [17,18,19].

Reaction temperature had a stronger impact on the frequency response compared to homogenization variables (Figure 1C). Emulsions processed at 55 °C displayed systematically higher G′ and G″ values compared to those prepared at 25 °C and 40 °C. In addition, a progressive attenuation of the low-frequency inflection in G′ was observed with increasing temperature, particularly at 55 °C. This behavior reflects enhanced chitosan–vanillin crosslinking and reduced structural relaxation at long deformation time scales, consistent with the formation of a denser and more persistent inter-droplet network [12,20].

Chitosan concentration produced the most pronounced changes in the frequency spectra (Figure 1D). Emulsions prepared at concentrations ≥ 0.8% (*w*/*v*) exhibited substantially higher viscoelastic moduli across the entire frequency range compared to lower concentrations, together with a progressive attenuation of the low-frequency inflection in G′. This reduced frequency dependence would indicate the development of a more interconnected and dynamically persistent droplet–polymer network, in which droplet mobility and structural rearrangements are strongly constrained. Consequently, structural relaxation at long deformation time scales is diminished, consistent with the formation of a percolated network, as previously reported for chitosan- and gum-stabilized emulsions [20,23,24,25]. In contrast, emulsions containing 0.6–0.7% chitosan displayed more pronounced frequency dependence and greater separation between G′ and G″ at low frequencies, which would indicate weaker and more transient inter-droplet associations.

The vanillin-to-chitosan molar ratio also modulated the frequency-dependent viscoelastic response (Figure 1E) by influencing crosslinking efficiency within the droplet–polymer network. Maximum reinforcement was observed near the stoichiometric ratio (0.7), where both G′ and G″ reached their highest values and the frequency dependence was less pronounced. In contrast, vanillin-deficient (0–0.3) and vanillin-excess (1.3) systems exhibited lower moduli and greater sensitivity to frequency, indicating reduced network connectivity and enhanced structural relaxation. These behaviors are consistent with suboptimal imine formation under non-stoichiometric conditions, as previously reported for chitosan–vanillin crosslinked systems [12].

The incorporation of non-ionic surfactants (Tween^®^ 20 and Tween^®^ 60) significantly modified the frequency-dependent response (Figure 1F). In these systems, both G′ and G″ increased compared to the surfactant-free emulsion, and the characteristic low-frequency inflection observed in the absence of Tween was no longer evident. The resulting spectra exhibited a more uniform frequency dependence across the explored range, suggesting altered relaxation dynamics. This behavior is consistent with previous observations in mixed biopolymer–surfactant systems, where rapid interfacial adsorption and competitive displacement can modify droplet–polymer interactions and network organization [19,26]. Nevertheless, this apparent reinforcement under oscillatory conditions does not necessarily translate into enhanced resistance under steady shear (see Section 3.4).

Overall, frequency sweep measurements confirm that, although all emulsions exhibited predominantly viscous behavior (G″ > G′), their viscoelastic response was strongly modulated by formulation variables governing network formation and interfacial organization. In particular, the attenuation or suppression of low-frequency relaxation under optimal conditions—namely higher chitosan concentrations, elevated reaction temperature, and near-stoichiometric vanillin-to-chitosan ratios—would indicate the development of more persistent droplet–polymer networks. These findings highlight the key role of formulation-driven structural connectivity in controlling the dynamic microstructural behavior of emulsion templates intended for oleogel applications.

### 3.4. Steady Shear Flow Behavior

**Figure 2:**
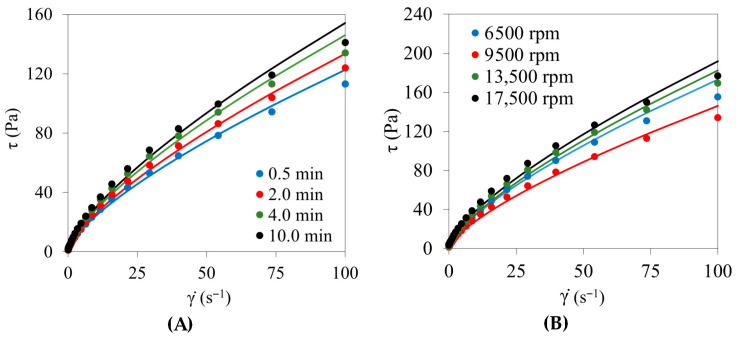

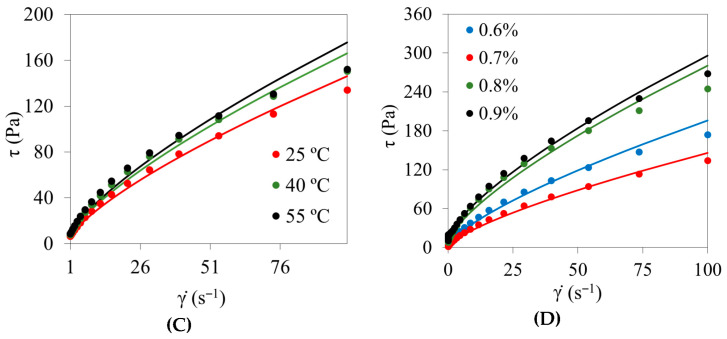

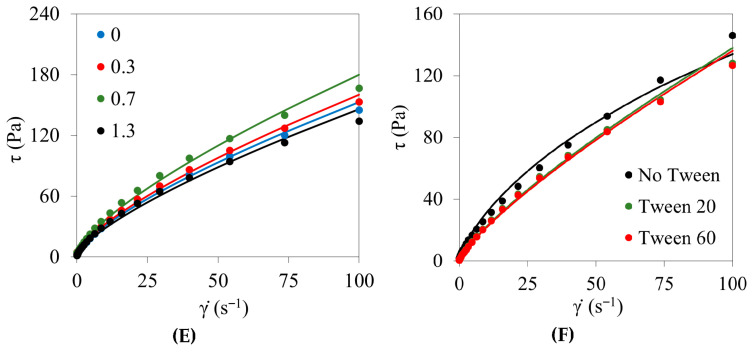
Effect of processing and formulation variables on the steady shear flow behavior of olive oil–water emulsions. Flow curves showing apparent viscosity as a function of shear rate; symbols represent experimental data and solid lines correspond to Herschel–Bulkley model fits. Unless otherwise specified, emulsions were prepared at 13,500 rpm, 25 °C reaction temperature, 0.8% chitosan, and vanillin-to-chitosan molar ratio 0.7. (**A**) Homogenization time (0.5, 2.0, 4.0, and 10.0 min); (**B**) homogenization speed (6500, 9500, 13,500, and 17,500 rpm); (**C**) reaction temperature (25, 40, and 55 °C); (**D**) chitosan concentration (0.6, 0.7, 0.8, and 0.9%); (**E**) vanillin-to-chitosan molar ratio (0, 0.3, 0.7, and 1.3); and (**F**) emulsifier type (no Tween, Tween^®^ 20, and Tween^®^ 60).

Steady shear measurements were performed to evaluate the flow behavior of the emulsions under continuous deformation and to assess their resistance to structural breakdown under processing-relevant shear conditions (Figure 2). Flow curves provide complementary information to oscillatory tests by probing the response beyond the linear viscoelastic regime, which is particularly relevant for pumping, mixing, and spreading operations [28].

All emulsions exhibited pronounced shear-thinning behavior over the investigated shear-rate range (Figure 2A–F), with apparent viscosity decreasing continuously as shear rate increased. Upward and downward sweeps were practically superimposable, confirming the absence of significant thixotropic effects, in agreement with the time sweep results (Section 3.1).

The experimental flow curves were well described by the Herschel–Bulkley model (Table 2), as confirmed by low RMSE values (0.004–0.137) and consistently high coefficients of determination (R^2^ > 0.98), indicating satisfactory agreement between experimental data and model predictions over the applied shear range. For surfactant-free systems, the flow behavior index (*n*) remained consistently below unity and remarkably stable across variables (*n* ≈ 0.73), confirming the shear-thinning behavior characteristic of structured emulsions [17,23,29]. This relative invariance of *n* suggested that formulation and processing conditions primarily modulated structural strength rather than altering the fundamental flow mechanism.

Yield stress (*τ*_0_) and consistency index (*K*) were the most sensitive parameters. Processing variables related to homogenization time (Figure 2A) produced moderate variations in *τ*_0_, ranging from 0.92 to 1.27 Pa, while *K* increased slightly from 4.20 to 5.30 Pa·s^n^. Similarly, homogenization speed (Figure 2B) induced gradual increases in *τ*_0_ (1.27–3.44 Pa) and *K* (5.02–6.54 Pa·s^n^), particularly at 17,500 rpm, which may be associated with improved dispersion during homogenization.

In contrast, formulation variables exerted much stronger effects. Increasing reaction temperature (Figure 2C) led to systematic increases in yield stress, from 1.27 Pa at 25 °C to 2.71 Pa at 55 °C, accompanied by higher consistency indices (5.02–5.99 Pa·s^n^), indicating enhanced chitosan–vanillin crosslinking and network cohesion [12,20].

Chitosan concentration (Figure 2D) produced the most pronounced changes. While systems at 0.6–0.7% exhibited *τ*_0_ values of 1.27–2.21 Pa, a sharp increase was observed at ≥0.8%, reaching 8.99 Pa (0.8%) and 14.21 Pa (0.9%), together with a marked rise in *K* (up to 9.76 Pa·s^n^). This abrupt enhancement confirms the formation of a percolated droplet–polymer network capable of sustaining finite stresses before yielding [20,23,24,25].

The vanillin-to-chitosan molar ratio (Figure 2E) modulated yield stress through crosslinking efficiency. Maximum *τ*_0_ was observed at the stoichiometric ratio (0.7; 2.34 Pa), whereas lower (0.0–0.3) and higher (1.3) ratios resulted in reduced values (1.11–2.05 Pa), consistent with suboptimal imine formation under non-ideal stoichiometric conditions [12].

In contrast, the incorporation of non-ionic surfactants (Figure 2F) dramatically reduced flow resistance. Yield stress decreased from 1.27 Pa in the surfactant-free system to 0.37 and 0.36 Pa for Tween^®^ 20 and Tween^®^ 60, respectively, representing a reduction of approximately 70%. Simultaneously, the flow behavior index increased to 0.79–0.80, indicating a shift toward less shear thinning behavior. This divergence between oscillatory reinforcement (Section 3.3) and steady shear softening highlights the competitive displacement of the chitosan–vanillin interfacial network by surfactant molecules, as reported in mixed biopolymer–surfactant systems [19,26].

Overall, steady shear measurements confirm that yield stress and consistency index are robust descriptors of emulsion structuring and processability. The sharp increase in τ_0_ above 0.8% chitosan and its systematic enhancement with reaction temperature underscore the central role of network connectivity in controlling flow resistance, whereas the strong reduction induced by surfactants reveals the limitations of purely interfacial stabilization strategies when structural integrity under shear is required.

### 3.5. Temperature Ramps and Thermal Stability

**Figure 3:**
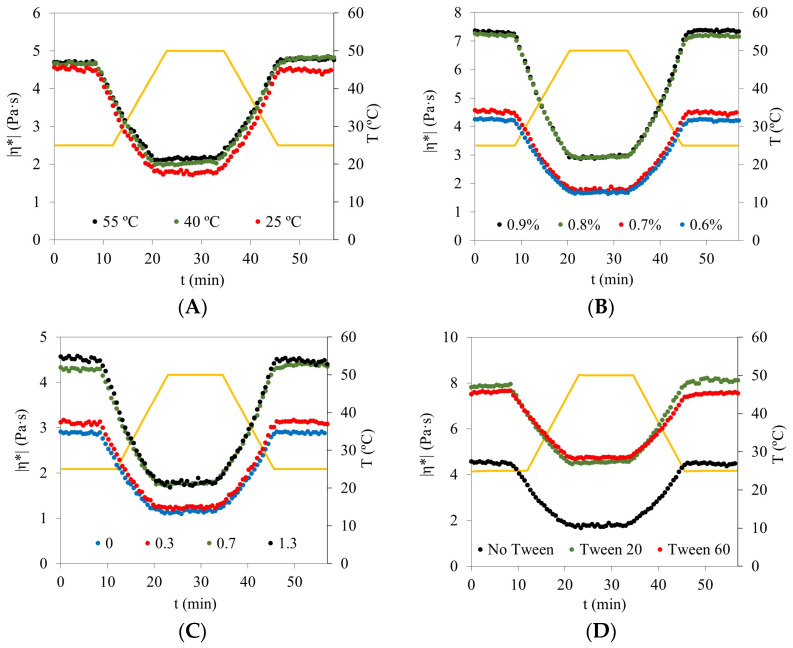
Temperature ramp profiles of olive oil–water emulsions under oscillatory conditions (1 Hz, 1% strain). Complex viscosity (|η*|) was monitored during heating (25–50 °C) and subsequent cooling. Unless otherwise specified, emulsions were prepared at 13,500 rpm, 25 °C reaction temperature, 0.8% chitosan, and vanillin-to-chitosan molar ratio 0.7. (**A**) Effect of reaction temperature (25, 40, and 55 °C); (**B**) effect of chitosan concentration (0.6, 0.7, 0.8, and 0.9%); (**C**) effect of vanillin-to-chitosan molar ratio (0, 0.3, 0.7, and 1.3); and (**D**) effect of emulsifier type (no Tween, Tween^®^ 20, and Tween^®^ 60).

Temperature ramp tests were performed under oscillatory conditions (1 Hz, 1% strain) to evaluate the thermal stability of emulsions prepared under different processing and formulation conditions (Figure 3). The evolution of complex viscosity (|η*|) during heating (25–50 °C) and subsequent cooling provides insight into the persistence of the droplet–polymer network under thermally induced stress [23,28].

Across all systems (Figure 3A–D), |η*| decreased progressively during heating, reaching a minimum near 50 °C, followed by partial or complete recovery upon cooling to 25 °C. This behavior reflects reversible thermal softening of the continuous phase and weakening of intermolecular interactions at elevated temperature.

The reaction temperature employed during emulsion preparation strongly influenced thermal resilience (Figure 3A). Emulsions reacted at 55 °C and 40 °C exhibited higher initial |η*| values (approximately 4.7–4.8 and 4.6–4.7 Pa·s, respectively) compared to those prepared at 25 °C (~4.4–4.5 Pa·s). Upon heating, viscosity decreased to ~2.1–2.2 Pa·s (55 °C), ~2.0 Pa·s (40 °C), and ~1.7–1.8 Pa·s (25 °C), followed by nearly complete recovery for the systems prepared at elevated temperatures. This enhanced recovery is consistent with more extensive chitosan–vanillin crosslinking at higher preparation temperatures [12,20].

Chitosan concentration exerted the most pronounced effect on thermal behavior (Figure 3B). Emulsions containing 0.8–0.9% chitosan displayed markedly higher initial |η*| values (~7.0–7.3 Pa·s) than those with 0.6–0.7% (~4.2–4.6 Pa·s). During heating, high-concentration systems decreased to ~3.0 Pa·s, whereas lower concentrations reached ~1.7–1.8 Pa·s. Upon cooling, emulsions formulated at ≥0.8% recovered almost completely, while those at ≤0.7% showed less complete structural recovery, confirming that percolated droplet–polymer networks possess greater resistance to thermally induced weakening [20,23,24,25].

The vanillin-to-chitosan molar ratio also modulated thermal response (Figure 3C). Systems prepared at the stoichiometric ratio (0.7) and at 1.3 exhibited higher initial |η*| (~4.3–4.5 Pa·s) compared to vanillin-deficient formulations (0–0.3; ~3.0–3.2 Pa·s). During heating, the optimal ratio retained higher viscosity values (~1.8–2.0 Pa·s at 50 °C), and recovery upon cooling was more complete, consistent with improved network connectivity under near-stoichiometric crosslinking conditions [12].

The incorporation of non-ionic surfactants further modified the thermal response (Figure 3D). Tween^®^ 20 and Tween^®^ 60 systems showed substantially higher initial |η*| (~7.5–8.0 Pa·s) than surfactant-free emulsions (~4.5 Pa·s). Although viscosity decreased during heating in all cases, Tween-containing emulsions maintained higher values (~4.5–5.0 Pa·s at 50 °C) and exhibited nearly complete recovery after cooling, particularly for Tween^®^ 20. Despite this enhanced oscillatory stiffness, it should be noted that surfactant-containing systems showed reduced yield stress under steady shear (Section 3.4), highlighting the distinct structural mechanisms governing oscillatory and continuous deformation responses [19,26].

Overall, temperature ramp measurements confirm that variables promoting stronger droplet–polymer connectivity—namely higher reaction temperature, increased chitosan concentration, and optimal vanillin stoichiometry—also enhance thermal resilience. The strong correspondence between viscoelastic reinforcement (Section 3.3), increased yield stress (Section 3.4), and improved thermal recovery demonstrates that network connectivity is the primary determinant of structural persistence in these emulsion templates intended for oleogel applications.

Based on this protocol, structured olive oleogels with consistent and reproducible properties were successfully obtained in our previous studies [30,31,32]. These studies highlight that optimization of the emulsion-preparation protocol is a critical step, since the initial droplet organization and interfacial interactions established during emulsification—together with the subsequent chitosan–vanillin Schiff-base reaction—govern network formation. Accordingly, as reported in those previous works, the combined effect of interfacial structuring and, in this case, covalent crosslinking determines the architecture, mechanical strength, and overall performance of the resulting oleogels.

## 4. Conclusions

The rheological characterization demonstrated that all chitosan–vanillin emulsions at Φ = 0.52 exhibited a predominantly viscous behavior (G″ > G′), consistent with concentrated emulsions below the jamming transition. A characteristic inflection in the G′ profile around 0.1 Hz, associated with a maximum in the damping factor, was observed in all systems except the Tween^®^-stabilized emulsions, suggesting the presence of transient droplet associations that were attenuated by crosslinking or by interfacial saturation. Steady shear measurements revealed overlapping upward and downward flow curves, indicating the absence of thixotropic and rheopectic effects. Moreover, the Herschel–Bulkley model adequately described the flow behavior of all formulations, consistently capturing their shear-thinning character.

Taken together, these results identify an optimal preparation protocol consisting of 4 min of homogenization at 9500 rpm, 0.9% *w*/*w* chitosan, a vanillin-to-chitosan molar ratio of 0.7, and reaction at 55 °C. This combination yielded emulsions with increased consistency and more pronounced rheological structuring, supporting their potential use as precursors of vegetable-oil oleogels.

While additional validation through complementary analytical approaches would be desirable to reinforce the robustness of these findings and improve the mechanistic interpretation, the proposed protocol was successfully applied to the preparation of olive oil oleogels. In this sense, it provides a solid initial basis for the design of related emulsified systems using other oils and gelling agents, and may be of practical value for other research groups working in this area.

## Figures and Tables

**Table 1:** Experimental variables and tested levels for the formation of olive oil–water emulsions stabilized by chitosan and vanillin.

Experimental Variable	Levels
Homogenization time (min)	0.5; 2.0; 4.0; 10.0
Homogenization speed (rpm)	6500; 9500; 13,500; 17,500
Reaction temperature (°C)	25; 40; 55
Chitosan concentration (% *w*/*v*)	0.6, 0.7, 0.8, 0.9, 1.0
Vanillin to chitosan molar ratio	0.0; 0.3; 0.7; 1.3
Emulsifier type	Tween^®^ 20; Tween^®^ 60

**Table 2:** Herschel–Bulkley, Equation (1), parameters of emulsions performed at different homogenization time/speed, reaction temperature, chitosan concentration, vanillin/chitosan molar ratio, and emulsifiers. *τ*_0_ = yield stress; *K* = consistency index; *n* = flow behavior index.

Variable	Condition	*τ*_0_ (Pa)	*K* (Pa·s^n^)	*n*	RMSE
Homogenization time (min)	0.5	0.94 ± 0.06 ^c^	4.20 ± 0.15 ^a^	0.73	0.087
	2.0	0.92 ± 0.04 ^a^	4.60 ± 0.16 ^b^		0.004
	4.0	1.27 ± 0.05 ^b^	5.02 ± 0.18 ^c^		0.081
	10.0	1.24 ± 0.05 ^ab^	5.30 ± 0.19 ^c^		0.071
Homogenization speed (rpm)	6500	1.64 ± 0.11 ^c^	5.92 ± 0.21 ^b^	0.73	0.121
	9500	1.27 ± 0.05 ^a^	5.02 ± 0.18 ^a^		0.081
	13,500	1.82 ± 0.07 ^b^	6.27 ± 0.22 ^bc^		0.059
	17,500	3.44 ± 0.14 ^d^	6.54 ± 0.23 ^c^		0.072
Reaction temperature (°C)	25	1.27 ± 0.05 ^a^	5.02 ± 0.18 ^a^	0.73	0.081
	40	2.61 ± 0.10 ^b^	5.67 ± 0.20 ^a^		0.087
	55	2.71 ± 0.11 ^b^	5.99 ± 0.21 ^b^		0.101
Chitosan concentration (% *w*/*w*)	0.6	2.21 ± 0.08 ^b^	6.70 ± 0.24 ^b^	0.73	0.078
	0.7	1.27 ± 0.05 ^a^	5.02 ± 0.18 ^a^		0.081
	0.8	8.99 ± 0.34 ^c^	9.41 ± 0.33 ^c^		0.097
	0.9	14.21 ± 0.56 ^d^	9.76 ± 0.34 ^c^		0.137
Vanillin/chitosan molar ratio (-)	0.0	1.11 ± 0.05 ^a^	4.80 ± 0.17 ^a^	0.73	0.116
	0.3	2.05 ± 0.08 ^b^	5.90 ± 0.21 ^b^		0.108
	0.7	2.34 ± 0.09 ^c^	6.50 ± 0.23 ^c^		0.113
	1.3	1.78 ± 0.07 ^b^	5.15 ± 0.18 ^a^		0.081
Emulsifier	None (baseline)	1.27 ± 0.05 ^b^	5.02 ± 0.18 ^b^	0.73	0.081
	Tween 20^®^	0.37 ± 0.02 ^a^	3.56 ± 0.12 ^a^	0.79	0.052
	Tween 60^®^	0.36 ± 0.01 ^a^	3.41 ± 0.12 ^a^	0.80	0.061

Superscript letters (a,b,c,d) denote ANOVA groupings (*p* < 0.05); same letter = no significant difference between conditions within each variable.

## Data Availability

The original contributions presented in this study are included in the article. Further inquiries can be directed to the corresponding author.
